# Severity of Fontan-Associated Liver Disease Correlates with Fontan Hemodynamics

**DOI:** 10.1007/s00246-020-02291-5

**Published:** 2020-02-01

**Authors:** Anastasia Schleiger, Madeleine Salzmann, Peter Kramer, Friederike Danne, Stephan Schubert, Christian Bassir, Tobias Müller, Hans-Peter Müller, Felix Berger, Stanislav Ovroutski

**Affiliations:** 1 Department of Congenital Heart Disease/Pediatric Cardiology, German Heart Center Berlin, Augustenburger Platz 1, 13353 Berlin, Germany; 2 Department of Pediatric Radiology, Charité-Universitätsmedizin Berlin, Berlin, Germany; 3 Department of Gastroenterology and Hepatology, Charité Universitätsmedizin Berlin, Berlin, Germany; 4 DZHK (German Center for Cardiovascular Research), Partner Site Berlin, Berlin, Germany; 5 Division of Cardiology, Department of Pediatrics, Charité Universitätsmedizin Berlin, Berlin, Germany

**Keywords:** Fontan-associated liver disease, Liver cirrhosis, Fontan surveillance, Fontan hemodynamics, Risk factors for FALD

## Abstract

Fontan-palliated patients are at risk for the development of Fontan-associated liver disease (FALD). In this study, we performed a detailed hemodynamic and hepatic assessment to analyze the incidence and spectrum of FALD and its association with patients' hemodynamics. From 2017 to 2019, 145 patients underwent a detailed, age-adjusted hepatic examination including laboratory analysis (FibroTest^®^, *n* = 101), liver ultrasound (*n* = 117) and transient elastography (FibroScan^®^, *n* = 61). The median patient age was 16.0 years [IQR 14.2], and the median duration of the Fontan circulation was 10.3 years [IQR 14.7]. Hemodynamic assessment was performed using echocardiography, cardiopulmonary exercise capacity testing and cardiac catheterization. Liver ultrasound revealed hepatic parenchymal changes in 83 patients (70.9%). Severe liver cirrhosis was detectable in 20 patients (17.1%). Median liver stiffness measured by FibroScan^®^ was 27.7 kPa [IQR 14.5], and the median Fibrotest^®^ score was 0.5 [IQR 0.3], corresponding to fibrosis stage ≥ 2. Liver stiffness values and Fibrotest^®^ scores correlated significantly with Fontan duration (*P*_1_ = 0.013, *P*_2_ = 0.012). Exercise performance was significantly impaired in patients with severe liver cirrhosis (*P* = 0.003). Pulmonary artery pressure and end-diastolic pressure were highly elevated in cirrhotic patients (*P*_1_ = 0.008, *P*_2_ = 0.003). Multivariable risk factor analysis revealed Fontan duration to be a major risk factor for the development of FALD (*P* < 0.001, OR 0.77, CI 0.68–0.87). In the majority of patients, hepatic abnormalities suggestive of FALD were detectable by liver ultrasound, transient elastography and laboratory analysis. The severity of FALD correlated significantly with Fontan duration and impaired Fontan hemodynamics. A detailed hepatic assessment is indispensable for long-term surveillance of Fontan patients.

## Introduction

The Fontan operation was introduced in 1968 by François M. Fontan as surgical palliation of tricuspid atresia [1]. Despite various modifications concerning surgical techniques, it has remained the palliative standard procedure for patients with univentricular physiology. With advances in surgical techniques, perioperative management, diagnostic capacities and adequate patient selection, early- and long-term survival of Fontan-palliated patients has improved over the past few decades [2–4]. Since the majority of Fontan patients reaches adulthood, there is growing evidence of end-organ dysfunction, especially affecting the liver [5, 6]. Fontan-associated liver disease (FALD) includes all abnormalities in liver structure and function which result from the non-physiological Fontan circulation: liver fibrosis, liver cirrhosis and hepatocellular carcinoma [7–10]. The etiology of FALD seems to be multifactorial, involving hypoxic and ischemic liver insults before and during the multi-step surgical palliation pathway for patients with univentricular anatomy [11]. The hallmarks of the Fontan circulation—diminished cardiac output and chronic central venous congestion—augment the structural and functional alteration of the liver: impaired hepatic vein drainage, sinusoidal dilatation and hemorrhagic necrosis result in the development of perisinusoidal and pericentral fibrosis leading to FALD [11]. Although various studies focus on the detection of FALD, hepatic assessment is not routinely included in Fontan surveillance, and risk factors for FALD development have not yet been identified.

In this study we performed a detailed hemodynamic and hepatic assessment to examine the incidence and spectrum of FALD and to analyze its association with hemodynamic parameters determined by echocardiography, cardiopulmonary exercise capacity testing and cardiac catheterization.

## Patients and Methods

### Patients

From July 2017 to July 2019, 145 of 475 (30.5%) Fontan patients treated in our institution underwent a detailed, age-adjusted hepatic assessment including liver ultrasound and laboratory analysis in patients > 7 years of age, and additional transient elastography in patients > 14 years. Hemodynamic assessment consisted of echocardiography and cardiopulmonary exercise testing. Cardiac catheterization was performed in patients with a clinical indication for an invasive evaluation of hemodynamics, e.g. clinical deterioration, impaired exercise capacity or decreased oxygen saturation. Two patients who received the Fontan operation as adults and died from early Fontan failure were excluded from further analysis. In patients with elevated liver enzymes and bilirubin, other causes for liver disease, such as infectious hepatitis, were ruled out. In our analysis, FALD was defined as the combination of elevated liver enzyme or bilirubin levels and abnormal liver ultrasound findings in accordance with reports of other investigators [12, 13]. Following the findings of Horowitz et al*.* [14] and Moon et al. [15], the combination of heterogeneous echotexture, surface nodularity, lobar atrophy or hypertrophy, and splenomegaly defined ultrasonographic diagnosis of liver cirrhosis. The study was approved by the institutional review board and the institutional ethics committee (decision number EA2/127/16). Informed consent was obtained from all individual participants included in the study.

### Laboratory Assessment

Liver function was evaluated using the liver enzymes alanine aminotransferase (ALT), aspartate aminotransferase (AST), γ-glutamyltransferase (γGT), total bilirubin, α_2_-macroglobulin, apolipoprotein A_1_ and haptoglobin. The laboratory parameters alanine aminotransferase, γ-glutamyltransferase, total bilirubin, α_2_-macroglobulin, apolipoprotein A_1_ and haptoglobin were measured in accordance with recommendations required by BioPredictive (Paris, France) to calculate biomarker fibrosis scores with FibroTest^®^. FibroTest^®^ was computed on the BioPredictive website (www.biopredictive.com). The calculated Fibrotest^®^ score was converted to liver fibrosis stage according to the METAVIR histological classification for liver biopsies [16].

### Liver Ultrasound and Transient Elastography

Following our protocol for hepatic Fontan surveillance, patients between 7 and 18 years of age underwent liver ultrasound by an experienced pediatric radiologist, while patients older than 18 years were referred to an experienced hepatologist. Liver ultrasound was performed following a multidisciplinary-developed protocol including measurement of liver and spleen size, examination of liver parenchyma and liver vein morphology, signs of portal hypertension, and Doppler sonography of liver vessels. Transient elastography (TE, FibroScan^®^, Echosens, France) was performed using a size M transducer in Fontan patients older than 14 years. A median value of 10 measurements of the right lobe of the liver was calculated. In patients with abnormal abdominal anatomy, e.g. situs inversus or heterotaxy, transient elastography was not considered diagnostically conclusive. Liver stiffness was reported in kilopascals (kPa).

### Hemodynamic Assessment

Hemodynamic assessment was performed following our protocol for Fontan surveillance and included echocardiography and cardiopulmonary exercise capacity testing. Echocardiographic systolic function of the single ventricle was graded as normal/mildly, moderately or severely impaired according to visual assessment of ventricular contractility performance. The degree of atrioventricular (AV) valve regurgitation was classified as none/mild, moderate or severe referring to the width of the colour Doppler regurgitation jet. Functional cardiopulmonary capacity testing was performed following a standardized institutional protocol using a cycle ergometer. Exercise was continued to the point of patients' maximum tolerance. Maximal oxygen uptake (VO_2_ max) was measured in ml/kg/min and normalized in % of predicted VO_2_ max using age-, gender- and body dimension-adjusted normative values suggested by Cooper et al*.* [17]. Cardiac catheterization was performed in patients with a clinical indication for an invasive hemodynamic evaluation (s.a.). The standard catheterization protocol included measurement of pulmonary artery pressure (PAP), systemic ventricular end-diastolic pressure (EDP), liver vein pressure (LVP) and liver vein wedge pressure (LVWP). Transpulmonary gradient (TPG) was calculated by the difference between mean pulmonary artery pressure and pulmonary capillary wedge pressure.

### Statistical Analysis

Demographic and surgical data were obtained from the medical records of the German Heart Center Berlin/ Department of Pediatric Cardiology at Charité Universitätsmedizin Berlin. Data are expressed as median and interquartile range calculated as 75th minus 25th percentile. Fontan duration was defined as the time between the date of Fontan operation and end of follow-up. Correlation analysis was performed using the Spearman or Kruskal–Wallis *H* test. The impact of ventricular function and the degree of AV valve regurgitation on the development of FALD were analyzed using the chi-square test. Risk factors for the development of FALD were evaluated using logistic regression analysis. Variables with *P* < 0.1 were included in a multivariable regression model. Statistic analysis was performed using the SPSS statistical software program SPSS for Windows (version 23, IBM Corp., Armonk, NY, USA). A *P* value < 0.05 was considered significant.

## Results

### Patient Characteristics

Table 1 displays baseline patient characteristics of the total cohort. From July 2017 to July 2019, 145 Fontan-palliated patients received a detailed, age-adjusted hepatic assessment in our institution. The total cohort consisted of 80 pediatric (55.2%) and 65 adult patients (44.8%). The median patient age was 16.0 years [IQR 14.2], and median age at Fontan operation was 3.7 years [IQR 3.3]. The most common underlying morphologies are listed in Table 1 and consisted of tricuspid atresia (*n* = 45, 31.0%), double inlet left ventricle (*n* = 21, 14.5%) and hypoplastic left heart syndrome (*n* = 22, 15.2%). Left ventricular dominance was found in 95 patients (65.5%). Absent sinus rhythm was found in 41 patients (28.3%), 29 of whom required a permanent pacemaker. The median duration of Fontan circulation was 10.3 years [IQR 14.7]. The Fontan procedure was performed using extracardiac conduit in 109 patients (75.2%), intracardiac lateral tunnel in 22 patients (15.2%) and other surgical modifications in 14 patients (9.7%). Sixty-one patients (42.1%) received primary fenestration. In 29 patients the fenestration was closed by intervention after a median time of 1.6 years [IQR 2.4]. Two patients (1.4%) were diagnosed with plastic bronchitis and 12 patients (8.3%) with protein-losing enteropathy. At the time of investigation, one patient with plastic bronchitis and seven patients with protein-losing enteropathy were in stable remission.

**Table 1:** Patient characteristics

Demographic assessment	No. (%)/median [IQR]
Patient age (years)	16.0 (14.2)
Age at Fontan operation (years)	3.5 (3.3)
Duration of Fontan circulation (years)	10.3 (14.7)
Gender	
Male	71 (49.0)
Underlying anatomy	
Tricuspid atresia	45 (31.0)
Double inlet left ventricle	21 (14.5)
Hypoplastic left heart syndrome	22 (15.2)
Complex transposition of great arteries	11 (7.6)
Unbalanced AVSD	16 (11.0)
RV hypoplasia with PA/IVS	11 (7.6)
Other	19 (13.1)
Predominant ventricular morphology	
Left	95 (66.5)
Right	50 (34.5)
Fontan type	
Intracardiac lateral tunnel	22 (15.2)
Extracardiac conduit	109 (75.2)
Other	14 (9.7)
Fenestration	
Primary	61 (14.5)
Secondary	6 (4.1)
PLE	12 (8.3)

*AVSD* atrioventricular septal defect, *RV* right ventricle, *PA/IVS* pulmonary atresia with intact ventricular septum, *PLE* protein-losing enteropathy

### Laboratory Assessment

The results of laboratory analysis for the total cohort are listed in Table 2. According to age-adjusted standard values of our laboratory, alanine aminotransferase (ALT) was elevated in 31 patients, aspartate aminotransferase (AST) in 27 patients, γ-glutamyltransferase (γGT) in 115 patients and bilirubin in 32 patients. Thrombocytopenia was found in 37 patients. The median Fibrotest^®^ fibrosis score was 0.5 [IQR 0.3]. With regard to the Fibrotest^®^ calculation, fibrosis was staged F2 in the majority of our patients (*n* = 49; Table 2).

**Table 2:** Results from hemodynamic and hepatic assessment

Hepatic assessment	Median (IQR)	No. (%) abnormal
Laboratory analysis		
AST (U/l)	36.6 (13.5)	27/133 (20.3)
ALT (U/l)	34.7 (16.5)	31/133 (23.3)
γGT (U/l)	71.1 (43.0)	115/133 (86.5)
Bilirubin (mg/dl)	1.0 (0.6)	32/130 (24.6)
Thrombocytes (K/µl)	207.2 (87.0)	37/133 (27.8)
Fibrotest^®^		
F0		6/101 (5.9)
F1		8/101 (7.9)
F2		49/101 (48.5)
F3		24/101 (23.8)
F4		14/101 (13.9)
Liver ultrasound findings		
Hepatomegaly		17/117 (14.5)
Splenomegaly		38/117 (32.5)
Heterogeneous parenchymal echotexture		83/117 (70.9)
Lobar atrophy/hypertrophy		27/117 (23.1)
Liver vein dilatation		69/117 (59.0)
Abnormal liver vein architecture		32/117 (27.4)
Hyperechogenic lesions		10/117 (8.5)
Surface nodularity		18/117 (15.4)
Ascites		13/117 (11.1)
Transient elastography	27.7 (14.5)	
Hemodynamic assessment		
Impairment of ventricular function		
None/mild		122/145 (84.1)
Moderate		20/145 (13.8)
Severe		3/145 (2.1)
AV valve insufficiency		
None/mild		122/145 (84.1)
Moderate		21/145 (14.5)
Severe		2/145 (1.4)
PAP (mmHg)	12.0 (4.0)	
EDP (mmHg)	8.0 (3.8)	
TPG (mmHg)	3.5 (3.0)	
LVP (mmHg)	12.0 (5.0)	
LVWP (mmHg)	13.0 (4.0)	

*AST* aspartate aminotransferase, *ALT* alanine aminotransferase, *γGT* γ-glutamyltransferase, *PAP* pulmonary artery pressure, *EDP* end-diastolic ventricular pressure, *TPG* transpulmonary gradient, *LVP* liver vein pressure, *LVWP* liver vein wedge pressure

### Liver Ultrasound and Transient Elastography

Liver ultrasound revealed hepatic parenchymal changes in 83 patients (Table 2). The most common ultrasound findings were heterogeneous echotexture (*n* = 83), liver vein dilatation (*n* = 69), altered liver vein morphology (*n* = 32) and segmental hypertrophy or atrophy (*n* = 25). Hyperechogenic lesions were present in 10 patients, and sonographic signs of liver cirrhosis were detectable in 20 patients (17.1%). Median TE values were 28.0 kPA [IQR 13.7] in the pediatric and 27.6 kPa [IQR 14.5] in the adult population. Both values were well above pediatric and adult TE cutoff values of 6.8 kPa and 7.5 kPA for severe liver fibrosis suggested by Fitzpatrick et al. [18] and Stebbing et al. [19]. Liver stiffness values were significantly higher in patients with sonography-detectable liver cirrhosis than in patients without (*P* = 0.017). Fibrotest^®^ fibrosis score correlated mildly with liver stiffness values measured by Fibroscan^®^ (*P* = 0.045). No correlation was found between sonographic evidence of structural hepatic damage and Fibrotest^®^ fibrosis score (*P* = 1.297).

### Hemodynamic Assessment

Echocardiographic examination was available in 145 patients and revealed no or mild impairment of systolic function in 122 patients, moderate impairment in 20 patients and severe impairment in three patients (Table 2). AV valve insufficiency was graded none or mild in 122 patients, moderate in 21 patients and severe in two patients. No correlation was found between systolic ventricular function, the degree of AV valve insufficiency and the incidence and severity of FALD in the form of liver cirrhosis.

**Fig. 1:**
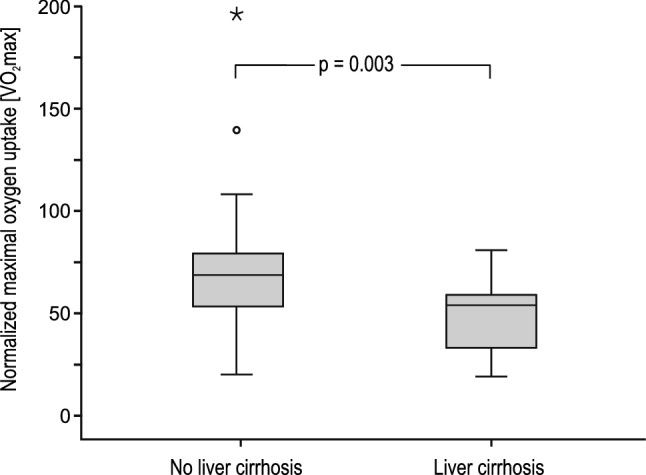
Relationship between exercise capacity (maximal oxygen uptake, VO_2_max) and liver cirrhosis. Data are shown as box plots representing two patient groups: patients with liver cirrhosis (*n* = 19) and without liver cirrhosis (*n* = 81). The top and the bottom of the rectangle indicate the 75th and 25th percentiles, and the middle horizontal line the median value. The vertical line extends from the maximal to the minimal VO_2_max value of each group

Exercise capacity testing was performed in 123 patients (84.8%). Median VO_2 _max was 25.6 ml/min/kg [IQR 8.5], equalling 64.8% [IQR 24.4] of age-, gender-, weight- and height-adjusted normative values. Normalized maximal oxygen uptake correlated strongly with the incidence of FALD (*P* = 0.012) and ultrasonography-detectable liver cirrhosis (Fig. 1; *P* = 0.003).

**Fig. 2:**
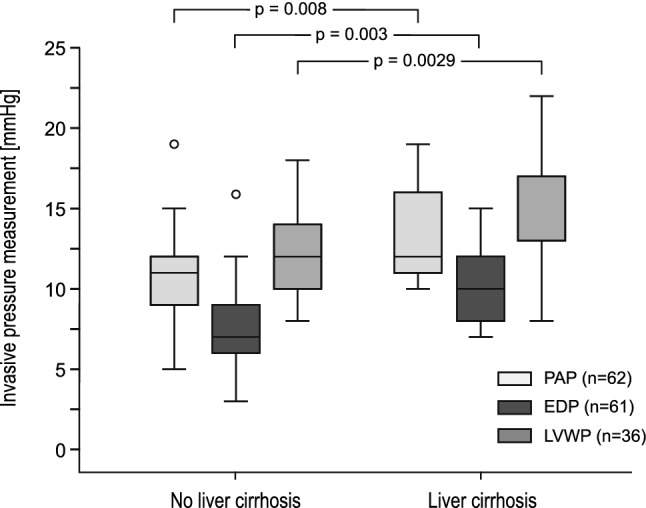
Correlation between invasively measured hemodynamic parameters and liver cirrhosis. Data are shown as box plots representing two patient groups: patients with and without liver cirrhosis. The top and the bottom of the rectangle indicate the 75th and 25th percentiles, and the middle horizontal line the median value. The vertical line extends from the maximal to the minimal invasively measured pressure in each group. Light gray box plots display values of pulmonary artery pressure of patients with liver cirrhosis (*n* = 14) and without liver cirrhosis (*n* = 48). Gray box plots with dashed black lines represent end-diastolic pressure of patients with liver cirrhosis (*n* = 14) and patients without (*n* = 47). Dark gray box plots display liver vein wedge pressure in cirrhotic (*n* = 9) and non-cirrhotic patients (*n* = 29). *PAP* pulmonary artery pressure, *EDP* end-diastolic pressure, *LVWP* liver vein wedge pressure

Seventy-six patients (53.1%) received invasive hemodynamic evaluation using cardiac catheterization; results are shown in Table 2. Invasive pressure measurement revealed a median PAP of 12.0 mmHg [IQR 4.0], EDP of 8.0 mmHg [IQR 3.8] and a median TPG of 3.5 mmHg [IQR 3.0]. Median liver vein pressure was 12.0 mmHg [IQR 5.0] and liver vein wedge pressure 13.0 mmHg [IQR 4.0]. PAP, EDP and LVWP were significantly higher in patients with sonography-detectable cirrhosis (*P*_1_ = 0.008, *P*_2_ = 0.003, *P*_3_ = 0.029; Fig. 2). TPG did not correlate with the presence of liver cirrhosis (*P* = 0.175). Liver vein wedge pressure correlated strongly with liver stiffness values measured by Fibroscan^®^ (*P*_1_ = 0.005). The presence of a primary fenestration had no statistically significant impact on the incidence of FALD (*P* = 0.138) or liver cirrhosis (*P* = 0.299).

### The Impact of Fontan Duration on Hemodynamics and FALD

**Fig. 3:**
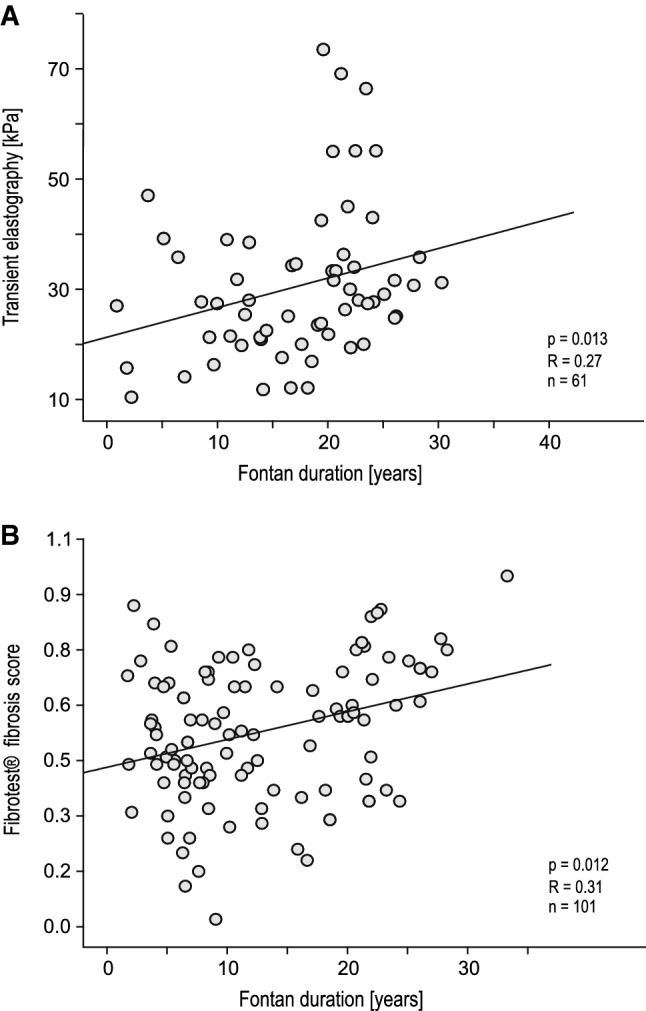
Scatter diagrams with regression lines representing the correlation between the duration of the Fontan circulation (ordinate) and liver stiffness values measured by transient elastography (abscissa, **a**) and Fibrotest^®^ fibrosis score (abscissa, **b**)

**Fig. 4:**
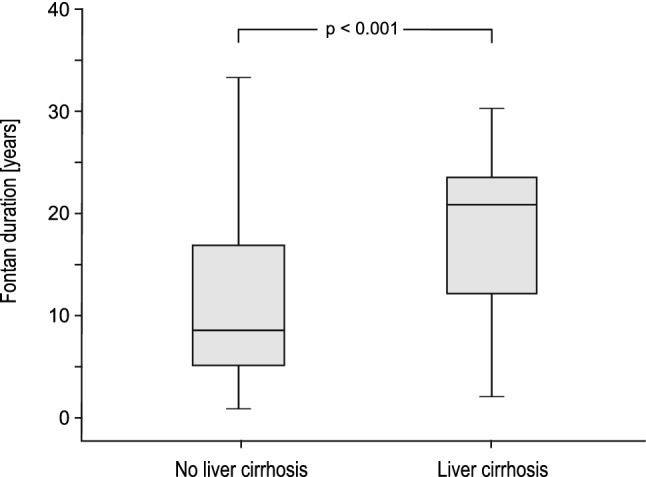
Correlation between the duration of the Fontan circulation and liver cirrhosis. Data are shown as box plots representing two patient groups: patients with liver cirrhosis (*n* = 20) and those without liver cirrhosis (*n* = 100). The top and the bottom of the rectangle indicate the 75th and 25th percentiles, and the middle horizontal line the median value. The vertical line extends from the maximal to the minimal time post Fontan of each group

In our cohort, Fontan duration showed a strong correlation with Fontan hemodynamics and hepatic abnormalities: pulmonary artery pressure significantly increased with the duration of the Fontan circulation (*P* = 0.026). A significant decrease in exercise capacity was detected with prolonged time post-Fontan (*P* < 0.001). Liver stiffness values measured by transient elastography and fibrosis score calculated by Fibrotest^®^ both correlated strongly with the duration of the Fontan circulation (*P*_1_ = 0.013, *P*_2_ = 0.012; Fig. 3). Fontan duration was significantly longer in patients with severe liver cirrhosis than in patients without (20.9 years [IQR 11.6] vs. 8.6 years [IQR 11.6], *P* < 0.001; Fig. 4).

### Risk Factor Analysis for the Development of FALD

Univariable risk factor analysis revealed Fontan duration, Fontan type and the absence of sinus rhythm to be significantly associated with FALD development (Table 3). Of the possible risk factors investigated in multivariable regression analysis, Fontan duration was found to be an independent risk factor for FALD (Table 3). Ventricular morphology did not influence FALD manifestation.

**Table 3:** Uni- and multivariable risk factor analysis for the development of FALD

Variable	Univariable analysis			Multivariable analysis		
	OR	95% CI	*P* value	OR	95% CI	*P* value
Systemic ventricle	0.83	0.35–2.0	0.681			
Fontan type	0.36	0.11–1.14	**0.082**	0.3	0.05–0.12	0.443
Sinus rhythm	0.24	0.06–0.41	**0.008**	0.21	0.08–0.26	0.298
Fontan duration	0.78	0.72–0.89	** < 0.001**	0.77	0.68–0.87	** < 0.001**

*OR* odds ratio, *CI* confidence interval

*P* values considered statistically significant are highlighted in bold

### Algorithm for Hemodynamic and Hepatic Fontan Surveillance

**Figs. 5–7:**
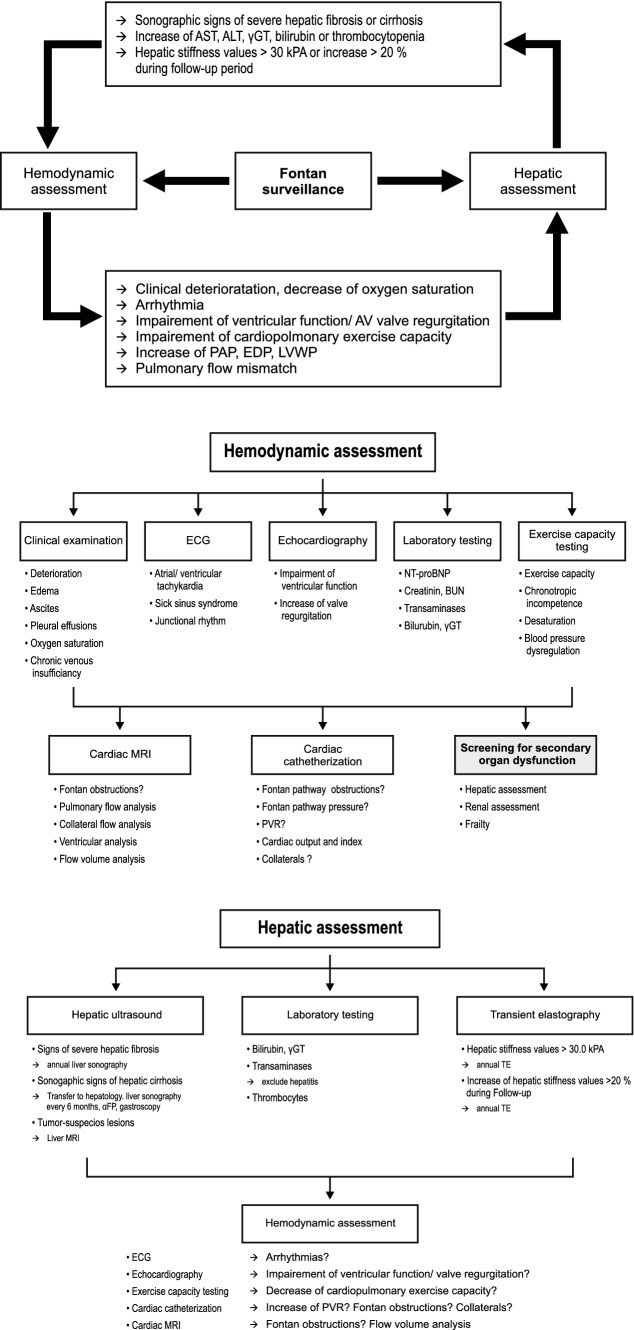
Algorithm for hemodynamic and hepatic assessment of Fontan-palliated patients. *AST* aspartate aminotransferase, *ALT* alanine aminotransferase, *γGT* γ-glutamyltransferase, *kPA* kilopascal, *PAP* pulmonary artery pressure, *EDP* end-diastolic ventricular pressure, *LVWP* liver vein wedge pressure, *ECG* electrocardiogram, *AV* atrioventricular, *BUN* blood urea nitrogen, *TE* transient elastography

Based on the findings presented in this study, we developed an algorithm for Fontan surveillance, which is demonstrated in Figs. 5, 6 and 7. We recommend annual or biannual referral of every Fontan-palliated patient to a specialized center for hemodynamic assessment including a detailed clinical examination, electro- and echocardiography, exercise capacity testing and laboratory analysis. In the case of clinical deterioration, cyanosis, reduced cardiopulmonary performance, arrhythmia, impaired ventricular function or increased AV valve regurgitation, a cardiac catheterization and/or MRI might be indicated for further hemodynamic evaluation. Additionally, a screening for second-organ diseases such as renal failure, frailty, chronic venous insufficiency and especially Fontan-associated liver disease should be performed.

According to our findings, in the majority of patients, the first hepatic abnormalities are evident 4 or 5 years after the Fontan operation. Therefore, we recommend a routine hepatic assessment including laboratory analysis (e.g. liver enzymes, bilirubin, thrombocytes) and liver ultrasound from the age of 7 years. Patients with a complicated course after the Fontan operation including early Fontan failure should receive routine hepatic surveillance before reaching 7 years of age. Patients with signs of liver cirrhosis should be referred to an experienced hepatologist and monitored closely for development of hepatocellular carcinoma or portal hypertension, including measurement of alpha-fetoprotein, liver sonography in 6-month intervals and performance of gastroscopy for diagnosis and treatment of gastroesophageal varices. In patients with tumor-suspicious lesions, a liver MRI is indicated.

Our findings underline the strong interrelation between Fontan hemodynamics and the development of Fontan-associated liver disease. Therefore, a detailed hepatic assessment should be performed when a patient presents with impaired hemodynamics (Figs. 5, 6). Conversely, progression of hepatic damage such as an increase in liver enzymes, sonographic signs of severe liver fibrosis or liver cirrhosis, or liver stiffness values > 30 kPA should prompt a detailed hemodynamic evaluation (Fig. 7).

Since every Fontan-palliated patient has a unique anatomy, this algorithm should only be considered as a recommendation for patients with an uncomplicated clinical course after Fontan operation and without signs of Fontan failure. The diagnostic and therapeutic approach should be individualized for each Fontan patient.

## Discussion

Fontan-associated liver disease is the most common second-organ dysfunction in Fontan-palliated patients, and is associated with increased morbidity and mortality [20–22]. This is the first study to perform a detailed, noninvasive hepatic assessment to examine the incidence and spectrum of FALD and to analyze its association with hemodynamic parameters determined by echocardiography, exercise capacity testing and cardiac catheterization.

Over the past few years, various invasive and noninvasive diagnostic instruments have been proposed for the detection of FALD, including liver biopsy, laboratory assessment, liver ultrasound, liver CT or MRI, and transient elastography [11–13, 23]. Although histopathological evaluation is considered the gold standard in the diagnosis of liver disease, in our study we focused on noninvasive assessment, considering that liver biopsy is associated with increased risk due to anticoagulation treatment and the unique vein anatomy of Fontan patients. Concerning long-term surveillance, noninvasive diagnostic instruments also appear to be more feasible and reproducible in the clinical setting.

### Noninvasive Diagnostic Instruments for the Detection of FALD

#### Transient Elastography

Transient elastography is considered a rapid and noninvasive diagnostic instrument to assess liver fibrosis by measuring liver stiffness [11]. Various studies report performing transient elastography in pediatric and adult Fontan patients with inconsistent results concerning correlation of TE values and Fontan duration, Fontan pressure or laboratory parameters [12–14]. In our cohort, a strong correlation was found between liver stiffness values and the duration of the Fontan circulation and liver fibrosis score calculated by Fibrotest^®^. TE values did not correlate with pulmonary artery pressure or EDP, suggesting that venous congestion does not mainly interfere with liver stiffness measurement. Moreover, in patients with sonographic detectable cirrhosis TE values were significantly higher than in patients without. These findings indicate TE might be useful to detect structural hepatic damage in Fontan patients. Nevertheless, normative liver stiffness values have to be validated for this particular patient cohort using laboratory tests and liver ultrasound.

#### Liver Ultrasound

Liver ultrasound is one of the most important diagnostic instruments for the detection of severe hepatic damage, such as liver cirrhosis. In our cohort, liver ultrasound revealed hepatic parenchymal changes in the majority of our patients. Severe liver cirrhosis was diagnosed based on sonographic scoring in 20 patients [14, 15]. Tumor-suspicious lesions were detected by liver ultrasound in three patients, but hepatocellular carcinoma was excluded by MRI. Based on its feasibility and reliability in the detection of hepatic damage liver, ultrasound should be performed in every Fontan patient during follow-up.

#### Laboratory Assessment

Laboratory assessment revealed an elevation of γGT in 86.5% of the total cohort. γGT is an enzyme located on the external surface of cellular membranes. The hallmarks of Fontan hemodynamics—low cardiac output and chronic venous congestion—may diminish vascular supply of intrahepatic bile ducts, leading to endothelial injury and release of γGT. Moreover, as a marker of increased oxidative stress, elevated γGT is associated with augmented risk of cardiovascular disease and heart failure [24]. γGT elevation is common in FALD but does not seem to correlate with its extent. In our cohort, the liver fibrosis score calculated by Fibrotest^®^ showed a strong correlation with the duration of the Fontan circulation, a mild correlation with liver stiffness values measured by Fibroscan^®^, but no correlation with liver ultrasound findings. Given these incoherent results, further studies are needed to evaluate the predictive ability of the Fibrotest^®^ fibrosis score with regard to liver fibrosis in Fontan patients.

All in all, our study demonstrates that one diagnostic instrument alone is ineffective in detecting Fontan-associated liver disease. Therefore, we propose the combination of laboratory analysis, transient elastography and liver ultrasound as basic hepatic screening for FALD in Fontan patients.

#### Impact of Fontan Hemodynamics on FALD

The development of Fontan-associated liver disease is based on a multi-step etiology including hypoxic insults, chronic venous congestion and low cardiac output [6]. This study revealed a high incidence of FALD among our patients based on laboratory and sonographic abnormalities found during examination. The severity of FALD varied between slightly elevated liver enzymes, minimal hepatic parenchymal changes and severe hepatic damage in the form of liver cirrhosis. In patients diagnosed with FALD, Fontan duration was significantly longer than in patients without (14.2 years [IQR 13.3] vs. 5.1 years [IQR 3.7]; *P* < 0.001). This significant correlation was also detected in patients with severe liver cirrhosis.

In our study, PAP, EDP and LVWP were not associated with the incidence of FALD, but correlated strongly with its severity. Cardiopulmonary exercise capacity was significantly decreased in patients diagnosed with both FALD and severe liver cirrhosis. Since PAP, EDP and LVWP exhibit a small variance and are not highly elevated in compensated Fontan patients compared with patients suffering from Fontan failure, a statistically significant increase might only become evident in patients with severe, but not mild, organ damage. In comparison, cardiopulmonary exercise testing seems to be more sensitive to detecting changes in Fontan hemodynamics. This might be explained by chronic venous congestion leading to diminished preload reserve in Fontan patients, which can be demonstrated by worsened cardiopulmonary performance. Our results confirm that impaired hemodynamics lead to severe hepatic damage. Conversely, these findings underline the importance of monitoring end-organ damage to detect impairment of Fontan hemodynamics. Further studies are needed to analyze and grade the severity of Fontan-associated liver disease and investigate the impact of hemodynamic changes during follow-up.

### Risk Factor Analysis

Univariable risk factor analysis revealed Fontan duration, Fontan type and absent sinus rhythm to be risk factors for FALD. The influence of Fontan type (intracardiac/lateral tunnel vs. extracardiac conduit) on FALD manifestation may reflect the change in surgical technique from late to early Fontan era, and underlines the importance of time post-Fontan in the development of end-organ damage.

The importance of atrial contraction in Fontan hemodynamics and the negative effect of arrhythmias and pacemaker dependency on long-term outcome in Fontan patients have already been described by other investigators [25]. Our results imply that absent atrial contraction leads to impaired atrial filling, reduced ventricular preload and diminished cardiac output. The consequences are elevated atrial and central venous pressure, which increases venous congestion and may aggravate the development of FALD.

In multivariable analysis, Fontan duration was found to be the strongest independent risk factor for FALD. These results underline the fact that FALD manifestation is inevitable. Consequently, a detailed hepatic assessment is mandatory in long-term surveillance of Fontan patients.

### Algorithm for Fontan Surveillance

Based on our findings, we developed a diagnostic algorithm for hemodynamic and hepatic Fontan surveillance. This algorithm highlights the strong interrelation between Fontan hemodynamics and the development of Fontan-associated liver disease: progression of hepatic damage should result in a detailed hemodynamic evaluation and vice versa.

#### Conclusion

Fontan-associated liver disease is the most common second-organ dysfunction in Fontan-palliated patients. In 70.9% of our patients, hepatic abnormalities suggestive of FALD were detectable by liver ultrasound, transient elastography and laboratory analysis. The incidence and spectrum of FALD correlate strongly with exercise capacity and Fontan hemodynamics. Fontan duration was found to be an independent risk factor for the development of FALD. FALD surveillance is imperative in the monitoring of Fontan-palliated patients.

### Limitations

There are several limitations to this study which should be noted. First, this is a prospective, cross-sectional study which includes many diagnostic instruments for hepatic and hemodynamic assessment. Therefore, not every patient received every diagnostic procedure mentioned, due to either death, noncompliance, movement, or missing indication for invasive procedures (e.g. cardiac catheterization). Second, we focused on noninvasive diagnostic instruments to detect Fontan-associated liver disease. Therefore, histological assessment to evaluate the extent and severity of liver alterations is missing. Third, although liver disease is common in Fontan patients, no universal definition of FALD exists, and further studies are needed to implement terminology and diagnostic and therapeutic algorithms. Fourth, risk factor analysis was limited by the number of patients included in the study.
